# Antipsychotic Drugs Alter Functional Connectivity between the Medial Frontal Cortex, Hippocampus, and Nucleus Accumbens as Measured by H215O PET

**DOI:** 10.3389/fpsyt.2012.00105

**Published:** 2012-12-06

**Authors:** Mark S. Bolding, David M. White, Jennifer A. Hadley, Martin Weiler, Henry H. Holcomb, Adrienne C. Lahti

**Affiliations:** ^1^Department of Psychiatry and Behavioral Neurobiology, The University of Alabama at BirminghamBirmingham, AL, USA; ^2^Department of Vision Sciences, The University of Alabama at BirminghamBirmingham, AL, USA; ^3^Department of Biomedical Engineering, The University of Alabama at BirminghamBirmingham, AL, USA; ^4^Maryland Psychiatric Research Center, University of Maryland School of MedicineBaltimore, MD, USA

**Keywords:** antipsychotic drugs, schizophrenia, functional connectivity, nucleus accumbens, hippocampus, medial frontal cortex

## Abstract

To evaluate changes in functional connectivity as a result of treatment with antipsychotic drugs (APDs) in subjects with schizophrenia (SZ), we identified a limited number of regions that have been implicated in the mechanism of action of APDs and that are part of a neuronal network known to be modulated by dopamine (DA). These regions consisted of the nucleus accumbens (NAcc), the hippocampus (Hip), and the medial frontal cortex (MFC). SZ participants were blindly randomized into a haloperidol treatment group (*n* = 12) and an olanzapine treatment group (*n* = 17). Using PET with 15O, we evaluated changes in functional connectivity between these regions during rest and task performance at three treatment time points: (1) at baseline, after withdrawal of all psychotropic medication (2 weeks), (2) after 1 week on medication, and (3) after 6 weeks on medication. Results from the two treatment groups were combined during analysis to investigate the common effects of APDs on functional connectivity. We found that the functional connectivity between MFC and NAcc significantly increased at week one, and then significantly decreased from week one to week 6. The functional connectivity between MFC and Hip significantly decreased at week one and week 6 relative to baseline. Critically, the strength of the functional connectivity between the MFC and Hip after 1 week of treatment was predictive of treatment response. This pattern of changes may represent an important biomarker for indexing treatment response. The regulation by APDs of the balance between prefrontal and limbic inputs to the striatum may be crucial to restoring adaptive behavior.

## Introduction

There are a number of antipsychotic drugs (APDs) available to treat schizophrenia (SZ). All current APDs are known to be dopamine D2 antagonists, and D2 blockade is believed to be a major part of their mechanism of action (Creese et al., 1976). First-generation APDs are characterized by predominant dopaminergic blockade, while second-generation APDs block both dopaminergic and serotoninergic receptors (Lahti et al., 1996). APDs primarily relieve the positive symptoms of SZ (such as hallucinations and delusions), and recent studies have shown that most first- and second-generation APDs alleviate positive symptoms with similar efficacy (Lieberman et al., 2005; McEvoy et al., 2006). Two meta-analyses have set out to characterize the time course of the response to APDs: one found that the greatest reduction of positive symptoms occurred in the first and second weeks of treatment, with a continued improvement over time afterwards (Agid et al., 2003), and the other found a linear reduction of positive symptoms over the course of, and up to 28 days of treatment (van den Oord et al., 2009). Both studies agree that the therapeutic response to APDs develops on a timescale of several weeks, but the mechanism of action of APDs is still poorly understood. This timescale implies an association between APD response and changes in neuronal connections. In this study, we investigated functional changes in neuronal connectivity using H215O positron emission tomography (PET).

Functional connectivity can be evaluated using H215O PET or functional magnetic resonance imaging (fMRI), and is defined as a correlation in activity between two different brain areas (Biswal et al., 1995). It has been repeatedly shown to reflect the underlying structural connectivity of the brain (Helmich et al., 2010), although it is variable and can exist between regions without direct structural links (van den Heuvel et al., 2009). A current theory of the pathophysiology of SZ is the dysconnection hypothesis, proposed by Friston and Frith (1995), which argues that the changes observed in SZ are due to abnormal interactions between brain areas. Lending support to this hypothesis, several studies have found differences in functional connectivity between persons with SZ and healthy controls (Schlosser et al., 2003; Meyer-Lindenberg et al., 2005; Liang et al., 2006; Garrity et al., 2007; Zhou et al., 2007; Whitfield-Gabrieli et al., 2009).

While SZ is known to alter functional connectivity, the effects of APDs on functional connectivity are not fully understood. Only two studies have evaluated functional connectivity changes in relation to APD treatment (Sambataro et al., 2009; Liu et al., 2010). Liu and colleagues used fMRI to evaluate changes in whole brain functional connectivity. Sambataro et al. used fMRI during task performance to examine functional connectivity changes within the default mode network. Neither study assessed differences in functional connectivity between an unmedicated time point and the early stages of treatment.

Our approach was to evaluate functional connectivity between a limited number of regions at three time points in treatment: before treatment, in the early stages of treatment, and after 6 weeks of treatment, at which time they were assumed to have reached a plateau in treatment response (van den Oord et al., 2009). The nucleus accumbens (NAcc), the hippocampus (Hip), and the medial frontal cortex (MFC) have been implicated in the mechanism of action of APDs (Berridge and Robinson, 1998). Our prior studies also suggest that these regions are involved in the mechanism of APD action (Lahti et al., 2006, 2009), and they are known to be part of a neural network modulated by dopamine (Kelley and Berridge, 2002), and therefore likely affected by APDs. The network defined by these regions is central to the model proposed by Goto and Grace (2008), which accounts for the role of DA in regulating the balance between limbic and prefrontal drives in the NAcc. Using *in vivo* electrophysiological recordings in the rat brain, Goto and Grace (2005) found that manipulations of DA released in the NAcc modulate hippocampal and cortical inputs; these experiments formed the basis of a model for how the disruption of DA can underlie a major psychiatric illness, such as SZ.

The model of Goto and Grace (2008) predicts APD treatment will alter the functional connectivity of MFC, NAcc, and Hip in specific ways. In particular, this model predicts that APD treatment should increase the functional connectivity between MFC and NAcc and decrease functional connectivity between MFC and Hip. Using PET with 15O, we tested the predictions of this model in patients before and after APD treatment. We chose to study a combination of patients treated with either a first or second generation APD, because we were interested in the changes to connectivity due to the drugs’ effects on the D2 receptors specifically (assumed to be common between first and second generation drugs). We evaluated the time course of regional cerebral blood flow (rCBF) patterns that developed after 1 and 6 weeks of treatment with either a first (haloperidol) or a second generation (olanzapine) APD in patients with SZ. Based on the model of Goto and Grace, we hypothesized that APD treatment would acutely increase the strength of the relationship between the MFC and NAcc. We further hypothesized that the magnitude of the change in functional connectivity between these regions would increase with increasing treatment duration, and that distinctive changes would be seen in the early (1 week) and later (6 week) stages of treatment.

## Materials and Methods

### Participants

This analysis includes resting-state imaging data previously reported in Lahti et al. (2009) and previously unpublished data acquired as part of the same project. Thirty-seven physically healthy individuals with SZ were recruited from the Residential Research Unit of the Maryland Psychiatric Research Center (MPRC) in Baltimore, MD, to participate in this study. Participants were selected from those who had been diagnosed with SZ by two independent research psychiatrists using DSM-IV criteria, based on the Structured Clinical Interview for DSM-III-R (SCID; Spitzer et al., 1992) and the patients’ clinical histories. All participants had been on stable doses of APDs; no patient had been treated with a long-acting APD depot preparation. Only patients deemed capable of understanding the risks of the study were selected to participate. All participants provided informed consent to a University of Maryland IRB-approved protocol. Details of the consent procedure can be found in Lahti et al. (2009).

Thirty-seven patients initially provided informed consent; however, eight dropped out during the study period [details of participant drop out were reported in Lahti et al. (2009)]. Participants ranged in age from 19 to 60 years, and duration of illness ranged from 1 to 44 years. The study cohort included 22 males and 7 females, and 8 Caucasians and 21 African Americans. All study participants remained in the inpatient research unit for the duration of the study. Participants were withdrawn from all psychotropic medications for 2 weeks before initiating PET scanning. This medication-free period allowed for the washout of APDs from central dopamine D2 receptors (Tamminga et al., 1993), and a baseline level of symptoms was evaluated for each participant using the Brief Psychiatric Rating Scale (BPRS, 1–7 scale; Overall and Gorham, 1962).

### Treatment

Following the medication washout period, study participants were blindly randomized into one of the following four groups: (1) 10 mg fixed dose of haloperidol for 6 days, followed by a clinically determined optimal dose of haloperidol (10–20 mg) for 5 weeks; (2) 12.5 mg fixed dose of olanzapine for 6 days, followed by a clinically determined optimal dose of olanzapine (12.5–25 mg) for 5 weeks; (3) placebo for 6 days followed by a clinically determined optimal dose of haloperidol (10–20 mg) for 5 weeks; or (4) placebo for 6 days followed by a clinically determined optimal dose of olanzapine (12.5–25 mg) for 5 weeks. Medications were prepared in similar-looking capsules by the hospital pharmacist. Optimal dose was determined by treating psychiatrists, who blindly adjusted medication in pre-determined increments (5 mg haloperidol, 6 mg olanzapine). Participants were placed on anticholinergic medication as treating psychiatrists deemed necessary. After the first week of the study, all patients were on an optimal dose of either haloperidol or olanzapine.

There were no statistically significant differences between treatment groups in age (38.3 ± 12.2 vs. 36.1 ± 10.5 years), length of illness (15.3 ± 14.1 vs. 11.3 ± 9.6 years), gender (male/female; 10/2 vs. 12/5), and race ratio (Caucasian/African American; 5/7 vs. 3/14). For details of treatment dosages, refer to Lahti et al. (2009).

Because we were specifically interested in changes to functional connectivity due to the common effects of APDs on D2 receptors, we combined treatment groups for all analyses. Our goal was to determine the common effect of APD treatment, as both first- and second-generation APDs have been found to decrease positive symptoms equivalently (Lieberman et al., 2005; McEvoy et al., 2006), and our previous study of this data reported similar rCBF changes in the MFC, NAcc, and Hip between medication groups (Lahti et al., 2009). Week one scans from participants receiving a placebo for the first 6 days were not included in our analysis, and, as treatment response has been shown to plateau after 4 weeks (van den Oord et al., 2009), patients from all treatment groups were deemed to have reached optimal response by week six of treatment.

### Experimental design

Participants were evaluated during three sessions over the course of treatment: (1) at baseline, before restarting APD treatment, (2) at 1 week, after 6 days of treatment, and (3) at 6 weeks, after 6 weeks of treatment. At each session, participants were scanned using H215O PET during three task conditions, as we were interested in changes in functional connectivity due to APDs that occurred independent of task (i.e., due to state not context). The H215O PET scans were then repeated during all task conditions, for a total of six H215O PET scans per session (two for each task condition per session). Additionally, participants’ symptoms were evaluated using the BPRS at each session.

### Experimental conditions

Participants received H215O PET scans during the following task conditions, which vary widely in terms of the networks they involve (Greicius et al., 2003): (1) at rest with eyes open, (2) during a sensorimotor control task, and (3) during an auditory discrimination task.

In the sensorimotor control task (SMC), participants were presented with 60 trials of either a high frequency tone (1500 Hz) or a low frequency tone (800–1492 Hz) of 100 ms duration. Participants were instructed to alternate between pressing a button held in their right hand and pressing a button held in their left hand when they heard the tone. Sixty tones were presented during each task. The inter-trial interval between the onset of the first tone and the onset of the next tone was 2 s.

The auditory discrimination task (DEC) was a graded error rate, forced choice task, described previously by Holcomb et al. (2000). In this task, participants were presented with 60 trials of either a high frequency tone (1500 Hz) or a low frequency tone (800–1492 Hz) of 100 ms duration. Participants were instructed to immediately press a button held in their right hand when they recognized a higher frequency tone or to press a button held in their left hand when they recognized a lower frequency tone. Sixty high and low frequency tones were presented during each task. The inter-trial interval between the onset of the first tone and the onset of the next tone was 2 s. Failure to respond was scored as an error, and the pitch difference was adjusted such that participants made the correct choice approximately 80% of the time. To control for potential changes in functional connectivity due to learning, participants were trained extensively on the DEC upon study enrollment (2 weeks prior to the first imaging session) until they consistently achieved the required performance accuracy. Participants were retrained immediately prior to each scanning session.

### Analysis of task performance

Task response accuracies and reaction times were compared between sessions using an analysis of variance (ANOVA) with Bonferroni correction for multiple comparisons.

### PET scanning

Participants were scanned on a GE Advanced 3D PET system, located at the PET Center of the Johns Hopkins Hospital. Each 90 s scan acquired 30 parallel slices with a center-to-center separation of 5 mm, an average transaxial resolution of 5 mm full width at half maximum (FWHM), and an average resolution of 6 mm FWHM in the center of the field of view. At the beginning of each session, a single 10 min transmission scan was acquired for attenuation correction using a 10 mCi 68 Ge rotating pin source. A 12 mCi H215O contrast bolus was then administered, using the method described by Raichle et al. (1983), without arterial blood sampling. Data acquisition began 15 s after bolus administration and continued for 90 s. Each scan resulted in one raw, three dimensional rCBF PET image corresponding to a specific task condition.

### PET image preprocessing

The raw PET rCBF images were preprocessed using statistical parametric mapping routines (SPM2; Friston et al., 1996) in the following manner:(1) all images from each participant were realigned to that participant’s first image, (2) all images were transformed into the standard Montreal Neurologic Institute (MNI) anatomical space, and (3), all images were spatially smoothed using a 12-mm full width at half maximum Gaussian kernel.

### Analysis of APD effects on rCBF

SPM8 was used to evaluate rCBF changes associated with APD treatment. Participants’ week one preprocessed rCBF PET images were compared to their baseline preprocessed rCBF PET images using a paired *t*-test to determine changes associated with 1 week of APD treatment. Participant’s week six preprocessed rCBF PET images were compared to their baseline preprocessed rCBF PET images using a paired *t*-test to determine changes associated with 6 weeks of APD treatment. Participant’s week six preprocessed rCBF PET images were compared to their week one preprocessed rCBF PET images using a paired *t*-test to determine changes between 1 and 6 weeks of APD treatment. Regions showing significant change with treatment were identified using *p* < 0.05, corrected using false discovery rate correction applied at the cluster-level.

### Analysis of APD effects on functional connectivity

Preprocessed rCBF PET images from all participants were grouped into four-dimensional (4D) vectors corresponding to each task condition at each session; preprocessed rCBF PET images were also combined into one “all tasks” condition for each session. This resulted in four 4D vectors from each session (12 vectors total).

Regions of interest (ROIs) were anatomically defined as masks in MNI space as follows: bilateral ROIs corresponding to the hippocampi were defined using the right and left hippocampus masks from the Automated Anatomical Labeling (AAL) atlas in PickAtlas (Maldjian et al., 2003); bilateral ROIs corresponding to the NAcc were hand traced in MNI space as described by Ballmaier et al. (2004); single ROI corresponding to the MFC was created by placing a 10-mm sphere at (−1, 47, −4), coordinates identified by Whitfield-Gabrieli et al. (2009). Additionally, a single mask of the whole brain was created using PickAtlas (29) to determine total intracranial activation.

Regions of interest masks were used to extract the first eigenvariate of the rCBF signal corresponding to that ROI from each of the 4D vectors. Functional connectivity between two ROIs was defined as the partial correlation between the rCBF signal eigenvariates from those ROIs, controlling for total intracranial activation, and was calculated for all conditions during all sessions. The resulting partial correlation coefficients (*r* scores) were transformed using Fisher’s r to *z* transformation into *z*-scores (Fox et al., 2005) for display and comparison.

SPSS was used to assess the effects of APDs on functional connectivity in the combined “all tasks” condition, such that if they showed correlated patterns of activity they were considered to be functionally connected. For each connection between two ROIs, the connection *z*-score from week one was compared to the connection *z*-score from baseline using a paired *t*-test to determine changes in functional connectivity associated with 1 week of APD treatment. Similarly, the connection *z*-score from week six was compared to the connection *z*-score from baseline and the connection *z*-score from week six was compared to the connection *z*-score from week one. Regions showing significant change with treatment were identified using the Hochberg-adjusted *p* < 0.05 (Benjamini and Hochberg, 1995).

### Association between treatment response and functional connectivity

Treatment response was defined to be the change in BPRS Psychosis Subscale score from baseline (before initiating treatment) to the end of the study (following 6 weeks of continuous APD treatment). To examine the relationship between treatment response and functional connectivity, the correlation between treatment response and functional connectivity (*z*-scores) was calculated for each session (at baseline, week one, and week six of treatment).

## Results

There were no significant differences between scanning sessions for response error rate or reaction time for the SMC or DEC (all *p* > 0.5). This suggests that any changes related to the tasks over time were not related to learning.

### APD effects on rCBF after 1 week of treatment

**Figure 1:**
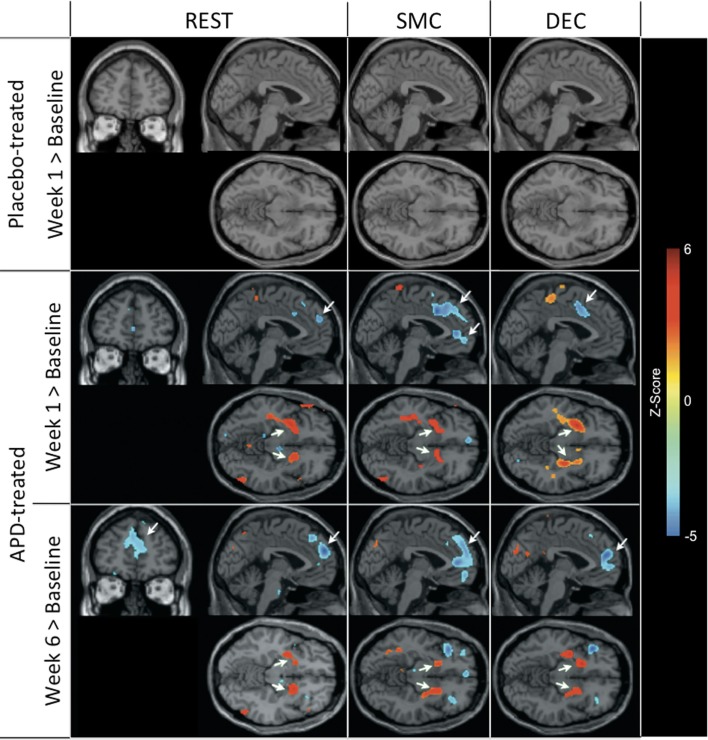
**rCBF changes relative to baseline with APD treatment after 1 and 6 weeks of treatment**. Increases in activation are shown in red and decreases are shown in blue. No significant changes were seen in the placebo-treated group at week 1 for any task. In the APD-treated group, there were significant increases in activation in NAcc after treatment with APDs (arrows, horizontal sections). There was a significant decrease in activation in medial frontal areas that shifted rostrally and dorsally between week 1 and week 6 of treatment (arrows, sagittal and coronal sections). The activation decrease was most significant during the sensory motor (SMC) and decision (DEC) task conditions. Coronal slices *y* = 47 mm and horizontal slices *z* = −10 mm (Montreal Neurological Institute coordinate system).

Figure 1 shows that, for all tasks, significant localized increases and decreases in rCBF relative to baseline were observed after 1 week of treatment. The most significant effects observed at week one were bilateral increases in rCBF in the striatum (cluster pFDR corrected < 0.001) and decreases in MFC. The clusters in the striatum were extensive and encompassed the NAcc and putamen. The peak activation was observed in the putamen. Activation peaks from each task condition are summarized in Tables 1– 3.

**Table 1:** **Rest condition**.

Region	Hemisphere	Cluster peak coordinates^a^	*P*^b^	*k*_E_
Rest rCBF increases following 1 week of APD treatment, SZ1 > SZ0				
Putamen	R	24, 14, 0	0.000	112
Putamen	L	−30, 4, −4	0.000	138
Rest rCBF increases following 6 weeks of APD treatment, SZ6 > SZ0				
Putamen	R	24, 8, −6	0.000	275
Putamen	L	−36, −6, 2	0.003	84
rCBF decreases following 6 weeks of APD treatment, SZ6 < SZ0				
Medial frontal gyrus	L, R	−2, 54, 26	0.003	76

*^a^Montreal neurological institute (MNI) anatomical coordinates in *x*, *y*, *z**.

*^b^*p* values FDR corrected*.

*SZ0, baseline; SZ1, after 1 week of antipsychotic drugs treatment; SZ6, after 6 weeks of antipsychotic drug treatment; APDs, antipsychotic drugs; R, right; L, left; kE, kernel extent in voxels; rCBF, regional cerebral blood flow*.

*Regional cerebral blood flow changes during rest from baseline after 1 and 6 weeks of treatment and between 1 and 6 weeks of treatment*.

**Table 2:** **Sensory motor task condition**.

Region	Hemisphere	Cluster peak coordinates^a^	*P*^b^	*k*_E_
SMC task rCBF increases following 1 week of APD treatment, SZ1 > SZ0				
Putamen	L	−32, 2, −4	0.002	108
SMC task rCBF increases following 6 weeks of APD treatment, SZ6 > SZ0				
Putamen	R	24, 4, −4	0.000	282
Putamen	L	−36, −2, −2	0.005	55

*^a^Montreal neurological institute (MNI) anatomical coordinates in *x*, *y*, *z**.

*^b^*p* values FDR corrected*.

*SZ0, baseline; SZ1, after 1 week of antipsychotic drugs treatment; SZ6, after 6 weeks of antipsychotic drug treatment; APDs, antipsychotic drugs; R, right; L, left; kE, kernel extent in voxels; rCBF, regional cerebral blood flow*.

*Regional cerebral blood flow changes during sensorimotor control task from baseline after 1 and 6 weeks of treatment and between 1 and 6 weeks of treatment*.

**Table 3:** **Decision task condition**.

Region	Hemisphere	Cluster peak coordinates^a^	*P*^b^	*k*_E_
DEC task rCBF increases following 1 week of APD treatment, SZ1 > SZ0				
Putamen	L	−26, 6, −6	0.000	646
Putamen	R	30, 14, −6	0.000	132
Putamen	R	28, 6, −6	0.021	41
DEC task rCBF decreases following 1 week of APD treatment, SZ1 < SZ0				
Anterior cingulate cortex	L, R	4, 10, 40	0.037	41
DEC task rCBF increases following 6 weeks of APD treatment, SZ6 > SZ0				
Putamen	L	−36, −4, −4	0.000	153
Putamen	R	24, 6, −2	0.000	150
Putamen	L	−18, 14, −8	0.006	60
DEC task rCBF decreases following 6 weeks of APD treatment, SZ6 < SZ0				
Medial frontal gyrus	L, R	0, 50, 18	0.011	53
DEC task rCBF increases between week 1 and week six of APD treatment, SZ6 < SZ1				
Putamen	L	−26, 4, −6	0.005	55

*^a^Montreal Neurological Institute (MNI) anatomical coordinates in *x*, *y*, *z**.

*^b^*p* values FDR corrected*.

*SZ0, baseline; SZ1, after 1 week of antipsychotic drugs treatment; SZ6, after 6 weeks of antipsychotic drug treatment; APDs, antipsychotic drugs; R, right; L, left; kE, kernel extent in voxels; rCBF, regional cerebral blood flow*.

*Regional cerebral blood flow changes during decision task from baseline after 1 and 6 weeks of treatment and between 1 and 6 weeks of treatment*.

### APD effects on rCBF after 6 weeks of treatment

Also seen in Figure 1 are localized changes in rCBF relative to baseline after 6 weeks of treatment. The most significant increases in rCBF were again observed in the striatum (cluster pFDR corrected < 0.001). As in the results at week one, the clusters were largely coextensive with NAcc and putamen. Significantly decreased rCBF was observed in MFC (cluster pFDR corrected < 0.001). The peak rCBF decreases were observed in the medial frontal gyrus. Deactivation was also observed in the medial temporal lobes bilaterally. Activation peaks from each task condition are summarized in Tables 1– 3.

### APD Effects on rCBF between 1 and 6 weeks of treatment

There were increases in rCBF in the thalamus at week six relative to week one (cluster pFDR corrected < 0.001) and decreases in rCBF in the putamen, MFC (cluster pFDR corrected < 0.001), and in the hippocampi (cluster pFDR corrected < 0.05).

**Figure 2:**
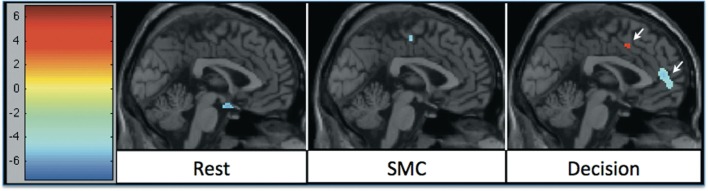
**rCBF changes relative to 1 week of APD treatment after 6 weeks of treatment**. Increases in activation are shown in red, and decreases are shown in blue. The significant decrease in activation in medial frontal areas shifted between week 1 and week 6 of treatment. The shift was most significant during the decision task condition.

In all tasks, APDs led to significantly decreased rCBF at both 1 and 6 weeks in the MFC. Importantly, there was a shift over time of the deactivation locus from the anterior cingulate to more rostral MFC. This deactivation shift was pronounced in the DEC task, as shown in Figure 2, where there was a relative increase in activation in the anterior cingulate, and a relative decrease in activation in MFC at week six relative to week one. Activation peaks from each task condition are summarized in Tables 1– 3.

### APD effects on functional connectivity

**Figure 3:**
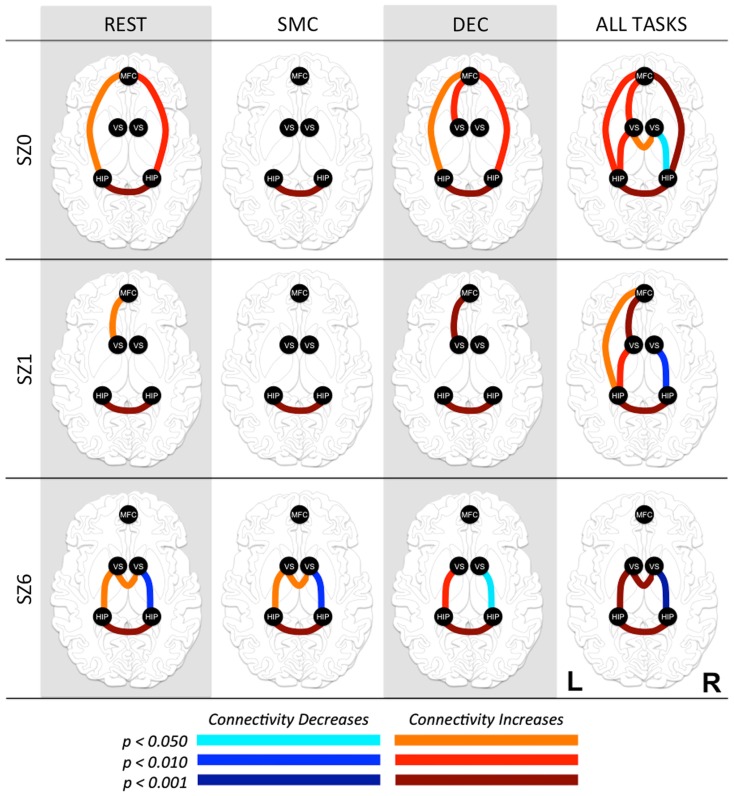
**Functional connectivity between regions of interest (ROI) at three time points in antipsychotic drug treatment (vertical axis) while performing three tasks and combined for all tasks (horizontal axis)**. Graph nodes represent ROI; graph edge color indicates the significance of the connection between nodes (*p*-value), for all connections with *p* < 0.05. SZ0, baseline; SZ1, 1 week of antipsychotic drugs treatment; SZ6, 6 weeks of antipsychotic drug treatment; HIP, hippocampus; VS; ventral striatum/nucleus accumbens; MFC, medial frontal cortex.

Pair-wise functional connectivity estimates between the MFC, the NAcc, and the Hip at baseline (off medication), after one and 6 weeks of treatment for each of the scan conditions (rest, SMC, and DEC), and for the combined scan conditions are shown in Figure 3. The coefficients of correlation are presented in Tables 4– 6 for the combined data, and in Tables 7– 9 for each of the individual conditions. For the combined tasks at baseline, there was significant functional connectivity between the MFC and the Hip bilaterally, between the NAcc and the Hip bilaterally, between the MFC and the left NAcc, and between the right and left NAcc. For the combined tasks at week one, there was significant functional connectivity between the MFC and left NAcc, between the NAcc and Hip bilaterally, and between the MFC and left Hip. For the combined tasks at week six, there was significant functional connectivity between the right and left NAcc and between the right and left Hip. A similar pattern of functional connectivity, albeit reduced, was observed across the individual task conditions. We observed that the left and right Hip had consistently high functional connectivity, supporting results previously reported by other groups (Stein et al., 2000). Significant functional connectivity between NAcc and the contralateral Hip was never observed.

**Table 4:** **Functional connectivity between regions of interest during all three task conditions at baseline**.

Area^a^	MFC	LNAcc	RNAcc	LHIP	RHIP
MFC	1.000				
LNAcc	0.339 *p* = 0.002**	1.000			
RNAcc	−0.018 *p* = 0.874	0.262 *p* = 0.018*	1.000		
LHIP	0.344 *p* = 0.002**	0.304 *p* = 0.006*	−0.115 *p* = 0.305	1.000	
RHIP	0.477 *p* = 0.000**	0.092 *p* = 0.414	−0.277 *p* = 0.012*	0.740 *p* = 0.000**	1.000

^a^Values reported are Pearson’s r and two-tailed significance, with 79 degrees of freedom. *p < 0.05, **p < 0.005. PFC, prefrontal cortex; LNAcc, left nucleus accumbens, RNAcc, right nucleus accumbens; LHIP, left hippocampus; RHIP, right hippocampus.

**Table 5:** **Functional connectivity between regions of interest during all three task conditions at week one**.

Area^a^	MFC	LNAcc	RNAcc	LHIP	RHIP
MFC	1.000				
LNAcc	0.573 *p* = 0.000**	1.000			
RNAcc	0.023 *p* = 0.869	0.249 *p* = 0.072	1.000		
LHIP	0.329 *p* = 0.016*	0.383 *p* = 0.005**	−0.163 *p* = 0.245	1.000	
RHIP	0.056 *p* = 0.692	0.122 *p* = 0.383	−0.400 *p* = 0.003**	0.716 *p* = 0.000**	1.000

^a^Values reported are Pearson’s r and two-tailed significance, with 51 degrees of freedom. *p < 0.05, **p < 0.005. PFC, prefrontal cortex; LNAcc, left nucleus accumbens, RNAcc, right nucleus accumbens; LHIP, left hippocampus; RHIP, right hippocampus.

**Table 6:** **Functional connectivity between regions of interest during all three task conditions at week six**.

Area^a^	MFC	LNAcc	RNAcc	LHIP	RHIP
MFC	1.000				
LNAcc	0.222 *p* = 0.054	1.000			
RNAcc	0.104 *p* = 0.373	0.391 *p* = 0.000**	1.000		
LHIP	0.068 *p* = 0.558	0.443 *p* = 0.000**	−0.057 *p* = 0.626	1.000	
RHIP	−0.126 *p* = 0.276	0.096 *p* = 0.411	−0.553 *p* = 0.000**	0.666 *p* = 0.000**	1.000

^a^Values reported are Pearson’s *r* and two-tailed significance, with 74 degrees of freedom. *p < 0.05, **p < 0.005. PFC, prefrontal cortex; LNAcc, left nucleus accumbens, RNAcc, right nucleus accumbens; LHIP, left hippocampus; RHIP, right hippocampus.

**Table 7:** **Functional connectivity between regions of interest during three task conditions at baseline**.

Task	Area^a^	MFC	LNAcc	RNAcc	LHIP	RHIP
Rest dF = 26	MFC	1.000				
	LNAcc	0.231 *p* = 0.238	1.000			
	RNAcc	−0.019 *p* = 0.924	0.265 *p* = 0.173	1.000		
	LHIP	0.416 *p* = 0.028*	0.248 *p* = 0.204	0.038 *p* = 0.848	1.000	
	RHIP	0.514 *p* = 0.005**	0.038 *p* = 0.847	−0.266 *p* = 0.171	0.700 *p* = 0.000**	1.000
Sensorimotor control task dF = 25	MFC	1.000				
	LNAcc	0.247 *p* = 0.213	1.000			
	RNAcc	−0.086 *p* = 0.669	0.295 *p* = 0.135	1.000		
	LHIP	0.188 *p* = 0.349	0.296 *p* = 0.134	−0.133 *p* = 0.508	1.000	
	RHIP	0.364 *p* = 0.062	0.069 *p* = 0.734	−0.216 *p* = 0.280	0.794 *p* = 0.000**	1.000
Decision task dF = 22	MFC	1.000				
	LNAcc	0.546 *p* = 0.006*	1.000			
	RNAcc	0.071 *p* = 0.743	0.247 *p* = 0.244	1.000		
	LHIP	0.442 *p* = 0.030*	0.378 *p* = 0.068	−0.260 *p* = 0.221	1.000	
	RHIP	0.572 *p* = 0.003**	0.192 *p* = 0.369	−0.378 *p* = 0.068	0.721 *p* = 0.000**	1.000

^a^Values reported are Pearson’s r and two-tailed significance. *p < 0.05, **p < 0.005.

*PFC, prefrontal cortex; LNAcc, left nucleus accumbens, RNAcc, right nucleus accumbens; LHIP, left hippocampus; RHIP, right hippocampus; dF, degrees of freedom*.

**Table 8:** **Functional connectivity between regions of interest during three task conditions at week one**.

Task	Area^a^	MFC	LNAcc	RNAcc	LHIP	RHIP
Rest dF = 16	MFC	1.000				
	LNAcc	0.517 *p* = 0.028*	1.000			
	RNAcc	−0.013 *p* = 0.959	0.265 *p* = 0.288	1.000		
	LHIP	0.137 *p* = 0.589	0.428 *p* = 0.077	−0.047 *p* = 0.852	1.000	
	RHIP	−0.032 *p* = 0.900	−0.093 *p* = 0.713	−0.395 *p* = 0.105	0.611 *p* = 0.007*	1.000
Sensorimotor control task dF = 14	MFC	1.000				
	LNAcc	0.447 *p* = 0.083	1.000			
	RNAcc	0.092 *p* = 0.736	0.262 *p* = 0.326	1.000		
	LHIP	0.461 *p* = 0.072	0.210 *p* = 0.436	−0.219 *p* = 0.415	1.000	
	RHIP	0.152 *p* = 0.575	0.141 *p* = 0.604	−0.425 *p* = 0.101	0.823 *p* = 0.000**	1.000
Decision task dF = 15	MFC	1.000				
	LNAcc	0.719 *p* = 0.001**	1.000			
	RNAcc	0. 038 *p* = 0.885	0.253 *p* = 0.326	1.000		
	LHIP	0. 385 *p* = 0.127	0.470 *p* = 0.057	−0.245 *p* = 0.343	1.000	
	RHIP	0.045 *p* = 0.862	0.280 *p* = 0.277	−0.400 *p* = 0.112	0.714 *p* = 0.001**	1.000

^a^Values reported are Pearson’s r and two-tailed significance. *p < 0.05, **p < 0.005.

*PFC, prefrontal cortex; LNAcc, left nucleus accumbens, RNAcc, right nucleus accumbens; LHIP, left hippocampus; RHIP, right hippocampus; dF, degrees of freedom*.

**Table 9:** **Functional connectivity between regions of interest during three task conditions at week six**.

Task	*Area^a^*	**MFC**	**LNAcc**	**RNAcc**	**LHIP**	**RHIP**
Rest dF = 24	MFC	1.000				
	LNAcc	0.296 *p* = 0.142	1.000			
	RNAcc	0.150 *p* = 0.142	0.438 *p* = 0.025*	1.000		
	LHIP	0.185 *p* = 0.367	0.428 *p* = 0.029*	−0.048 *p* = 0.817	1.000	
	RHIP	−0.039 *p* = 0.851	0.052 *p* = 0.799	−0.507 *p* = 0.008*	0.734 *p* = 0.000**	1.000
Sensorimotor control task dF = 24	MFC	1.000				
	LNAcc	0.105 *p* = 0.610	1.000			
	RNAcc	0.148 *p* = 0.470	0.463 *p* = 0.017*	1.000		
	LHIP	−0.168 *p* = 0.412	0.388 *p* = 0.050*	−0.105 *p* = 0.609	1.000	
	RHIP	−0.295 *p* = 0.144	0.034 *p* = 0.870	−0.579 *p* = 0.002**	0.629 *p* = 0.001**	1.000
Decision task dF = 20	MFC	1.000				
	LNAcc	0.352 *p* = 0.108	1.000			
	RNAcc	−0.116 *p* = 0.608	0.318 *p* = 0.149	1.000		
	LHIP	0.271 *p* = 0.223	0.542 *p* = 0.009*	0.023 *p* = 0.919	1.000	
	RHIP	0.126 *p* = 0.576	0.178 *p* = 0.428	−0.531 *p* = 0.011*	0.656 *p* = 0.001**	1.000

^a^Values reported are Pearson’s r and two-tailed significance. *p < 0.05, **p < 0.005.

*PFC, prefrontal cortex; LNAcc, left nucleus accumbens, RNAcc, right nucleus accumbens; LHIP, left hippocampus; RHIP, right hippocampus; dF, degrees of freedom*.

**Figure 4:**
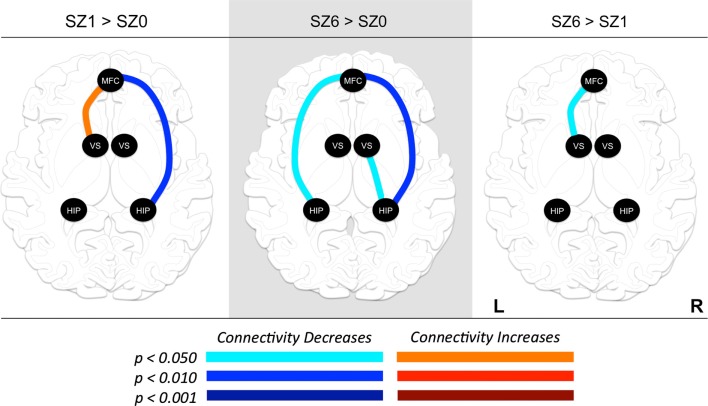
**Changes in functional connectivity between regions of interest (ROI) during combined tasks from baseline to week one of antipsychotic drug treatment (left), baseline to week six of antipsychotic drug treatment (center), and between weeks one and six of antipsychotic drug treatment (right)**. Graph nodes represent ROI; graph edge color indicates the significance of the connectivity change between nodes (p-value), for all connections with *p* < 0.05. SZ0, baseline; SZ1, 1 week of antipsychotic drugs treatment; SZ6, 6 weeks of antipsychotic drug treatment; HIP, hippocampus; VS; ventral striatum/nucleus accumbens; MFC, medial frontal cortex.

We observed the following changes in functional connectivity for the combined tasks over time (as illustrated in Figure 4): (1) after 1 week of treatment, the functional connectivity between MFC and the left NAcc increased relative to baseline (*p* < 0.05). The strong baseline connectivity between MFC and right Hip was reduced significantly (*p* < 0.05). (2) After 6 weeks of treatment, there was a significant decrease in functional connectivity between MFC and bilateral Hip (*p* < 0.05) relative to baseline, and a significant decrease in the functional connectivity between the right NAcc and ipsilateral Hip relative to baseline. (3) After 6 weeks of treatment, there were significant reductions in MFC-left NAcc functional connectivity relative to week one (*p* < 0.05). There was also a trend (*p* = 0.07) towards a significant decrease in the functional connectivity between the MFC and the left Hip. Significant changes in functional connectivity between time points are listed in Table 10.

**Table 10:** **Changes in functional connectivity between three time points in antipsychotic drug treatment**.

	SZ1 > SZ0	SZ6 > SZ0	SZ6 > SZ1
Medial frontal cortex to left nucleus accumbens			
*z*-score	1.65	−0.78	−2.32
Significance ^a^	0.0495*	0.2177	0.0102*
Medial frontal cortex to right nucleus accumbens			
*z*-score	0.23	0.75	0.44
Significance ^a^	0.4090	0.2266	0.3300
Medial frontal cortex to left hippocampus			
*z*-score	−0.09	−1.78	−1.49
Significance ^a^	0.4641	0.0375*	0.0681
Medial frontal cortex to right hippocampus			
*z*-score	−2.56	−2.84	−1.00
Significance ^a^	0.0052*	0.0023**	0.1587
Left nucleus accumbens to left hippocampus			
*z*-score	0.49	0.98	0.39
Significance ^a^	0.312	0.1263	0.348
Right nucleus accumbens to right hippocampus			
*z*-score	−0.77	−2.08	−1.08
Significance ^a^	0.2207	0.0188*	0.1401

^a^One-tailed significance. *p < 0.05, **p < 0.005.

*SZ0, baseline; SZ1, 1 week of antipsychotic drugs treatment; SZ6, 6 weeks of antipsychotic drug treatment*.

### Association between treatment response and functional connectivity

To quantify treatment response, the change in BPRS Psychosis subscale score from baseline to week six was calculated for each participant. This score was positively correlated with the functional connectivity between the MFC and left Hip (*r*^2^ = 0.219, *p* < 0.05) at 1 week of treatment, and negatively correlated with the functional connectivity between the right NAcc and right Hip (*r*^2^ = 0.109, *p* < 0.05) at 6 weeks of treatment. These results suggest that functional connectivity between MFC and left Hip may be predictive of treatment response.

## Discussion

The present study found that APDs alter the activation and functional connectivity between the MFC, NAcc, and Hip after 1 week of treatment, and that further changes unfold over the next 5 weeks. As predicted, the functional connectivity between MFC and NAcc significantly increased at week one. However, this was followed by a significant decrease in functional connectivity between these regions from week one to week six. The functional connectivity between MFC and Hip significantly decreased at week one and week six relative to baseline. Critically, the strength of the functional connectivity between MFC and Hip after 1 week of treatment was predictive of treatment response, explaining 21% of the variance in BPRS Psychosis subscale score changes between baseline and week six. Across a range of cognitive tasks, APDs significantly increased rCBF in striatum after 1 and 6 weeks of treatment. APDs also led to significantly decreased rCBF in the MFC at weeks one and six relative to baseline, with the deactivation locus shifting to more rostral and ventral MFC from week one to week six. Likewise, APDs led to significant rCBF changes in Hip.

### Functional connectivity

Functional connectivity, defined as a measure of the “statistical dependencies among remote neurophysiological events” (Friston et al., 2007), is increasingly used to investigate the organization of the brain. Compared to healthy controls, changes in functional connectivity in SZ have been reported by multiple groups (Lawrie et al., 2002; Schlosser et al., 2003; Meyer-Lindenberg et al., 2005; Liang et al., 2006; Bluhm et al., 2007; Zhou et al., 2007; Whitfield-Gabrieli et al., 2009; Lynall et al., 2010; Salvador et al., 2010). Notably, Salvador et al. (2010) reported an increased functional connectivity at rest between the MFC and the caudate in chronic, medicated individuals with SZ with respect to normal volunteers, and Meyer-Lindenberg et al. (2005) observed abnormal functional connectivity between the dorsolateral prefrontal cortex and the Hip in medication-free patients with SZ during performance of a working memory task. Our findings extend the work of Salvador and Meyer-Lindenberg and colleagues by showing that APDs modify the functional connectivity between MFC and Hip and between MFC and NAcc.

Prior to our investigation, only two groups have reported changes in functional connectivity with APD treatment (Sambataro et al., 2009; Liu et al., 2010). However, neither group investigated the acute changes in functional connectivity such as those found in this study. Lui et al. (2010) reported that 6 week APD treatment in APD-naïve individuals with SZ resulted in a widespread attenuation of functional connectivity, including between the caudate and the PFC, and between the PFC and the parahippocampus. Sambataro et al. (2009) reported increased connectivity between the ventromedial PFC and the rest of the default mode network after 8 vs. 4 weeks of treatment with olanzapine; however, the patients were not studied while medication-free. Thus, our study extends these findings by reporting both an acute increase and decrease in functional connectivity between the MFC and NAcc and between the MFC and Hip, respectively, in association with APD.

Relevant to the study of APDs, changes in functional connectivity of resting-state fMRI have also been observed in conjunction with the dopamine precursor, l-dopa (Kelly et al., 2009). Acute l-dopa administration in healthy participants increased functional connectivity between the NAcc and ventrolateral PFC, whereas diet-induced DA depletion perturbed fronto-striatal functional connectivity during performance of the Wisconsin Card Sorting Task (Nagano-Saito et al., 2008). In participants with Parkinson’s disease compared to healthy participants, there was a remapping of cerebral connectivity that reduced the spatial segregation between different cortico-striatal loops (Helmich et al., 2010). Thus, changes in fronto-striatal functional connectivity have been observed with both DA antagonists and agonists, and in situations of DA depletion either in Parkinson’s disease or diet-induced. These findings are additional evidence that manipulations of DA levels in the NAcc can have significant effects on functional connectivity such as the changes we observed.

### The MFC-NAcc-Hip network

**Figure 5:**
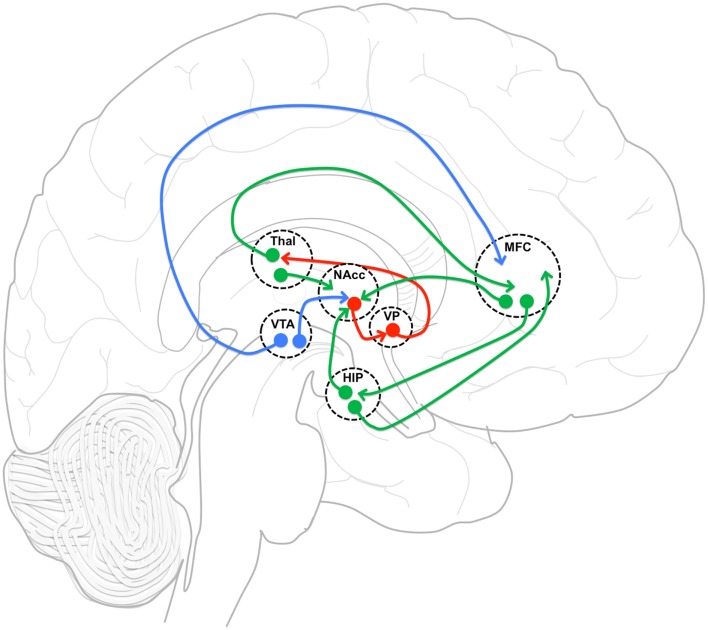
**Functional connectivity model**. Functional connectivity model of medial frontal cortex (MFC), and hippocampus (HIP) incorporating gabaergic (red), glutametergic (green) and dopaminergic (blue) projections with an emphasis on MFC and HIP projection interactions in nucleus accumbens (NAcc). VP, ventral pallidum; Thal, thalamus; VTA, ventral tegmental area.

The neuronal network examined in this study (Figure 5) includes the MFC, NAcc, and Hip, which have been implicated in the mechanism of action of APDs. Imaging studies have consistently demonstrated that first- and second-generation APDs increase functional activity in the striatum (Holcomb et al., 1996; Lahti et al., 2005, 2009). In addition, lower pre-treatment striatal metabolic rate (Buchsbaum et al., 1992) and greater post-treatment striatal activation have been shown to predict treatment response (Buchsbaum et al., 1992; Lahti et al., 2009). Likewise, changes in ACC (Ngan et al., 2002; Lahti et al., 2009) and hippocampal (Liddle et al., 2000; Medoff et al., 2001; Lahti et al., 2003, 2009) activation following first- and second-generation APD treatment have been reported and found to be correlated with or to predict response. Taken together, these studies point to the importance of the MFC-NAcc-Hip network in the treatment of subjects with SZ by APDs, and led us to investigate their functional connectivity.

The neuronal network defined by these regions is reciprocally connected and is thought to be essential in regulating the balance between limbic and prefrontal inputs in the service of goal-directed behaviors (Mogenson et al., 1980; Grace, 2000; Gruber et al., 2009). Goto and Grace (2008) have proposed that dopamine release in the NAcc modulates the balance between the MFC and Hip inputs, thus allowing adjustments in behavior in response to environmental demands. Upon testing this model, they showed that an acute perfusion of a DA D2 antagonist in the NAcc led to the facilitation of MFC over Hip evoked responses (Goto and Grace, 2007). As predicted by this acute animal model, we observed an increase in the strength of functional connectivity between MFC and NAcc after 1 week of treatment. Also acutely, we observed a decrease in functional connectivity between MFC and Hip. Most remarkably, the strength of the functional connectivity between MFC and Hip at week one was predictive of treatment response. We also observed changes after the first week that could not have been predicted based on the acute D2 antagonist model.

Because all APDs are known to act directly at D2 receptors, a primary site of APD action is believed to be the striatum. In the NAcc, glutamatergic (GLU) inputs from the PFC and the Hip, and DA inputs mainly originating from the ventral tegmental area (VTA) synapse on the same dendritic spines and shafts of medium sized GABA-ergic projection neurons (Kotter, 1994; Starr, 1995), creating a circuit that is ideal for the interaction of Glu and DA inputs, and their modulation by APDs. Therefore, we have hypothesized that one of the steps by which APD action is achieved is through changes in functional connectivity within this network, putatively restoring the balance between prefrontal and limbic inputs.

How could APDs induce changes in functional connectivity? Changes in long term plasticity (LTD and LTP) are thought to result from changes in the spines’ structure of GABA neurons via the interaction of DA and Glu (Wolf, 2003; Surmeier et al., 2007; Shen et al., 2008). Putatively, APDs, by modifying the balance between DA and Glu inputs at the level of the spines of GABA-ergic neurons, could affect spines’ structure and induce plastic changes affecting functional connectivity with other brain regions.

### Limitations and potential confounds

We did not study a healthy control group, so we were unable to evaluate whether APDs normalized the pattern of functional connectivity in the NAcc, Hip, MFC network.

A 2-week withdrawal is likely not enough to allow medication-induced brain changes to fully revert to a baseline condition. However, the potent rCBF increase observed in the dorsal and ventral striatum by APDs in this study strongly suggested that DA receptors were not blocked by residual medication.

We investigated the effect of APDs on a limited network of interconnected regions (MFC-NAcc-Hip). This model is based on a large body of clinical and preclinical data. The substantia nigra, which is an important part of the network discussed in this study, was not incorporated into the neural network, although we clearly observed changes in its function with treatment. Further studies are needed to investigate the role of the substantia nigra in the APD-induced changes that we observed.

## Conclusion

In order to evaluate changes in functional connectivity as a result of treatment with APDs, we identified a limited number of regions that have been implicated in the mechanism of action of APDs and that are part of a neuronal network known to be modulated by DA. Paralleling the time course of therapeutic response, we found that APDs altered functional connectivity between regions of this network after 1 week of treatment, and that further changes unfolded after the next 5 weeks of treatment. Critically, the strength of the functional connectivity between the MFC and Hip after 1 week of treatment was predictive of treatment response. This pattern of changes might represent an important biomarker for indexing treatment response. The regulation by APDs of the balance between prefrontal and limbic inputs to the striatum through plastic changes may be crucial to restoring adaptive behavior.

## Conflict of Interest Statement

Adrienne Lahti has received an investigator initiated grant from Pfizerand drug from Ortho-McNeil Janssen Scientific Affairs LLC, and served as a consultant for Lundbeck, Inc. Mark Bolding, David White, Jennifer Hadley, Martin Weiler, and Henry Holcomb have no financial interests to disclose.
